# Heart of the Matter: Decoding the Underdiagnosed Cardiac Amyloidosis

**DOI:** 10.7759/cureus.50527

**Published:** 2023-12-14

**Authors:** Michael E Kaiser, Toni-Ann J Lewis

**Affiliations:** 1 Internal Medicine, St. George's University School of Medicine, Brooklyn, USA; 2 Internal Medicine, New York-Presbyterian Brooklyn Methodist Hospital, New York, USA

**Keywords:** spect imaging, diastolic heart failure, pyp scan, attr amyloidosis, under-diagnosed, amyloid transthyretin, amyloid light chain proteins, toxic abnormally folded proteins, restrictive cardiomyopathy, cardiac amyloidosis

## Abstract

Cardiac amyloidosis, a rare disorder marked by toxic amyloid protein deposition in the myocardium, contributes significantly to restrictive cardiomyopathy. We present an 85-year-old female diagnosed with amyloid transthyretin (ATTR) cardiac amyloidosis, emphasizing the under-recognition of this condition.

The pathophysiology of cardiac amyloidosis involves misfolded protein accumulation, which impairs myocardial function. Differentiating AL and ATTR is crucial, with ATTR predominance. Diagnosis relies on echocardiography, cardiac magnetic resonance, nuclear imaging, and biomarker testing. A positive pyrophosphate (PYP) scan, compatible echocardiographic features, and the absence of systemic myeloma signs diagnose ATTR amyloidosis. Management includes heart failure treatment, arrhythmia control, and disease-modifying strategies like Tafamidis, Inotersen, and Patisiran. Genotyping guides prognostic and therapeutic considerations.

Recognizing cardiac amyloidosis as an underlying cause of heart failure with preserved ejection fraction necessitates collaboration between cardiology and hematology. Improved awareness, innovative diagnostics, and targeted therapies are crucial to reduce diagnostic delays and enhance outcomes.

## Introduction

Cardiac amyloidosis is a leading cause of restrictive cardiomyopathy marked by extracellular deposition of misfolded protein fragments known as amyloid [1]. The deposition of toxic abnormally folded proteins called amyloid is derived from two sources: amyloid light (AL) chain proteins and amyloid transthyretin (ATTR) [2]. Its diagnosis often requires a high degree of clinical suspicion alongside cardiovascular imaging and is one of the most under-diagnosed disease entities [3]. This paper highlights the pathophysiology of cardiac amyloidosis, stemming from the extracellular accumulation of misfolded proteins, ultimately impairing myocardial function. Differentiating between AL and ATTR types is essential, with ATTR being the predominant cause. Diagnosis is intricate, demanding an array of tools, including echocardiography, cardiac magnetic resonance, nuclear imaging, and biomarker testing. Clinical suspicion is pivotal due to the disease's nonspecific manifestations. Notably, a strongly positive pyrophosphate (PYP) scan, compatible echocardiographic features, and absence of systemic myeloma signs can reliably diagnose ATTR amyloidosis. Comprehensive management encompasses heart failure treatment, arrhythmia control, and disease-modifying strategies like Tafamidis, Inotersen, and Patisiran. Genotyping becomes crucial for prognostic and therapeutic considerations in ATTR amyloidosis. This case accentuates the significance of recognizing cardiac amyloidosis as an underlying cause of heart failure with preserved ejection fraction, prompting interdisciplinary collaboration between cardiology and hematology. Elevating awareness, employing innovative diagnostic methods, and implementing targeted therapies are crucial for reducing diagnostic delays and enhancing patient outcomes.

## Case presentation

**Figure 1:**
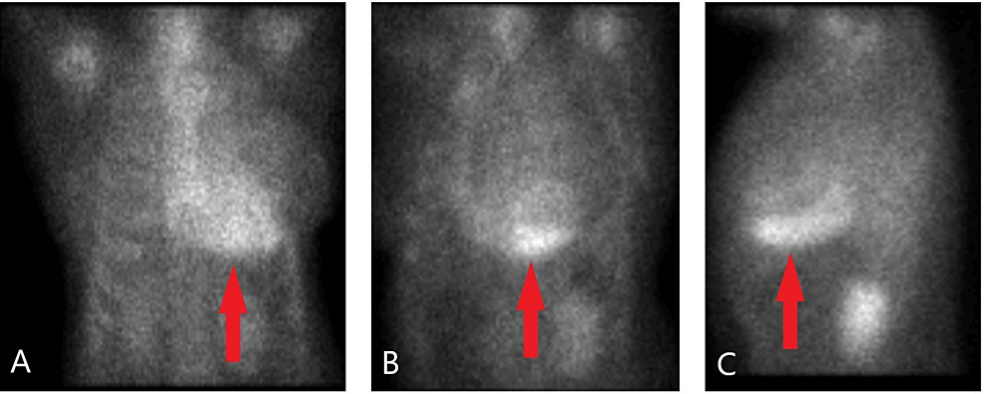
Planar technetium-labeled cardiac scintigraphy showing Grade 3 (severe uptake - heart greater than bone) (A) Anterior projection, (B) anterolateral projection, (C) lateral projection

**Figure 2:**
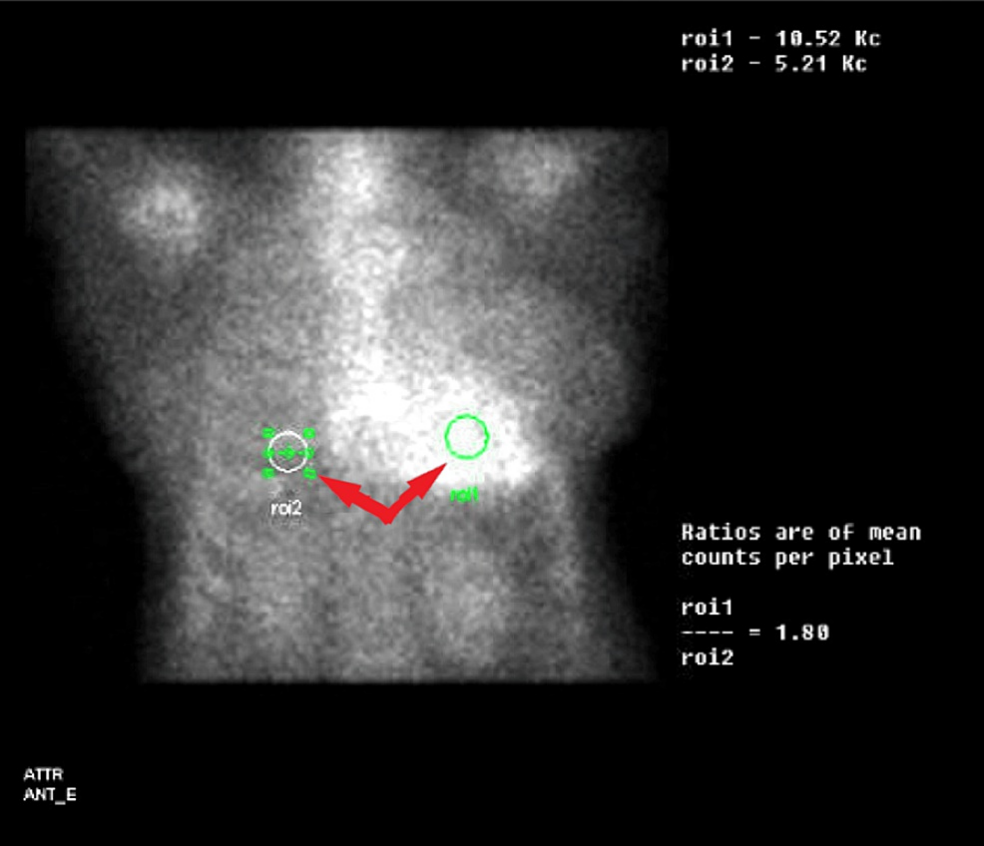
Planar semiquantitative evaluation showing heart to contralateral lung ratio (H/Cl) of 1.8

**Figure 3:**
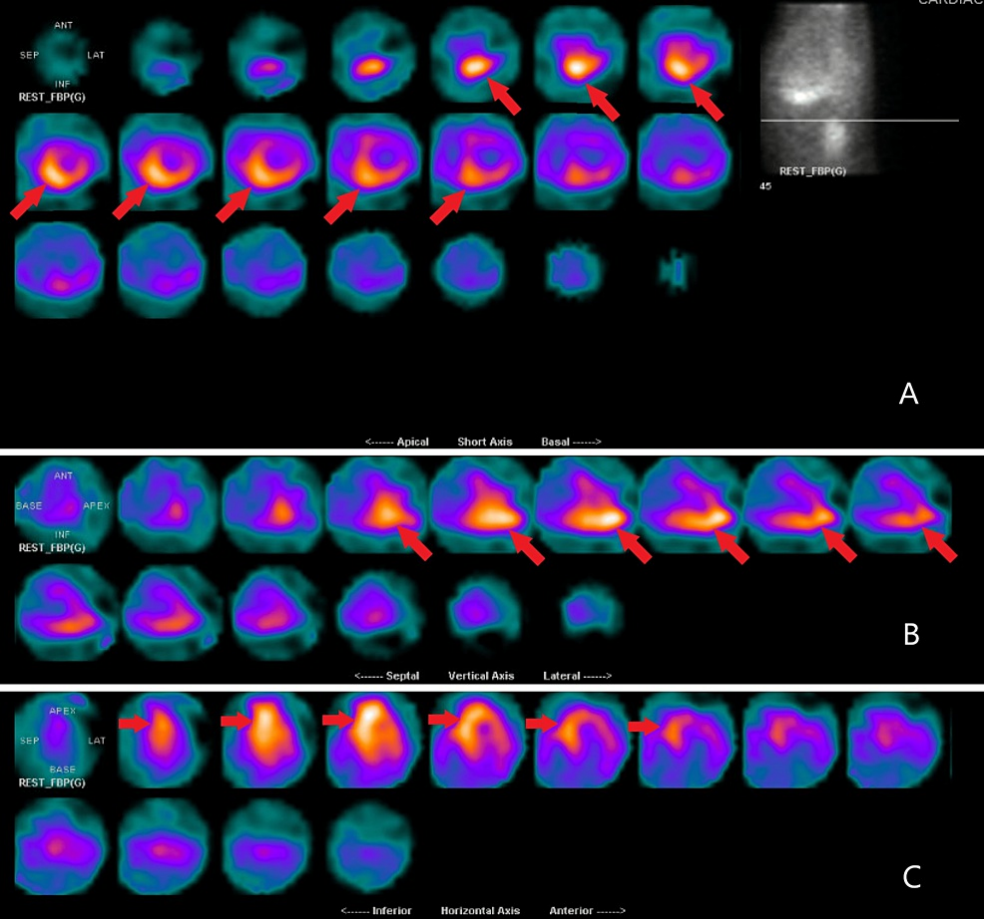
Supine SPECT/CT 99mTc-PYP imaging showing PYP uptake in the left ventricle (A) Short axis, (B) vertical axis, (C) horizontal axis Red Arrows: Focal concentration of 99mTc-PYP in myocardial tissue PYP - pyrophosphate

An 85-year-old female presented to the emergency department from her cardiologist office due to concerns from an abnormal echocardiogram finding. Her past medical history was significant for hypertension and dementia. The patient endorsed two pillow orthopnea and occasional lower extremity swelling relieved with Furosemide. However, she denied palpitations, chest pain, dyspnea at rest or on exertion. On admission she was afebrile with mild bradycardia at 59 bpm and hypertensive at 150/90 mmHg. Physical examination was unremarkable for signs of volume overload. Outpatient echocardiogram showed an ejection fraction of 55%-60% alongside normal left ventricular size, systolic function and wall motion. There was, however, mildly increased left ventricular wall thickness and Grade III diastolic dysfunction seen on imaging (Video 1). A left anterior fascicular block was seen on electrocardiogram and serology was significant for elevated proBNP but otherwise unremarkable as well. The results of cardiac 99mTc-scintigraphy showed Grade 3 - myocardial uptake greater than rib uptake with mild/absent rib uptake (Figures 1A-1C), planar semiquantitative evaluation showed heart to contralateral lung (H/CL) ratio of 1.8 (Figure 2), and SPECT/CT 99mTc-PYP imaging revealed PYP uptake in the myocardium (Figures 3A-3C). These findings prompted Hematology consultation, but workup for multiple myeloma was negative and systemic AL amyloidosis was ruled out. Patient was diagnosed with new onset heart failure with preserved ejection fraction due to TTR cardiac amyloidosis. She was discharged with instructions for outpatient TTR gene testing and directed to follow up with Hematology and Cardiology.

**Video 1:** Echocardiogram showing increased left ventricular wall thickness and Grade III diastolic dysfunction

## Discussion

Cardiac amyloidosis occurs due to the deposition of toxic abnormally folded proteins called amyloid into the extracellular space, leading to the stiffening of the myocardium and depressed cardiac function [1-4]. This rare disorder is one of the leading causes of restrictive cardiomyopathy and one of the most underdiagnosed disease entities [3]. The abnormally folded proteins derive from two sources: AL chain proteins - as seen in conditions such as multiple myeloma, and ATTR [3], with the most common being AL light chain proteins [3]. Transthyretin-related amyloid cardiomyopathy is slowly progressive and clinically well-tolerated [1]. Diagnosis is often delayed until marked ventricular wall thickening, profound diastolic dysfunction, and conduction disease have occurred [1]. Cardiac biopsy remains the gold standard for diagnosing amyloid cardiomyopathy. However, non-invasive diagnostic methods are also implemented [1]. This includes echocardiography with strain imaging, cardiac magnetic resonance (CMR)/nuclear imaging, electrocardiography (ECG), and serum biomarker testing, including BNP and troponin [1]. Once identified, genotyping is essential in patients with ATTR amyloidosis as more than 120 gene variants have been identified and are essential to predict treatment response and prognosis [3]. Diagnosis often requires high clinical suspicion as patients commonly present nonspecific findings such as dyspnea, fatigue, and edema [2]. A strongly positive bone tracer cardiac scintigraphy (myocardial uptake of more significant than bone Grade 2 or 3 or bone to the chest wall uptake ratio >1.5), echocardiographic features consistent with cardiac amyloidosis, and the absence of systemic signs and symptoms of multiple myeloma are almost diagnostic of TTR amyloidosis [3]. Treatment focuses on three areas: heart failure, management of arrhythmias, and initiation of disease-modifying agents (i.e., tafamidis, inotersen, patisiran) [2]. The disease-modifying agents used for treatment focus on three specific steps in ATTR production: (1) tafamidis inhibits the dissociation of TTR tetramers by binding the T4-binding sites, keeping unbound protein monomers from dissociating from the quaternary structure, which negates monomer misfolding [5], (2) inotersen inhibits the liver's production of both variant and wild-type TTR amyloid proteins [5], and (3) finally, patisiran is a small RNA molecule that blocks the expression of both wild-type TTR amyloid and variant [5]. These drugs work together to reduce disease progression and improve symptomology alongside the standard heart failure regimens.

The prognosis for untreated ATTR amyloidosis is variable. ATTR amyloidosis generally has a better prognosis than AL amyloidosis, progressing slowly and typically presenting in the seventh decade of life with an average survival time of 7-10 years if untreated [3]. Mutant ATTR has an overall four-year survival of 16%. The survival of mutant ATTR depends on the type of mutation. The Val30Met is the most common mutation in mutant ATTR, with an overall prognosis of 79%, whereas the Val122Ile mutation carries a four-year prognosis of 40% [6]. Our patient's mutation status was unknown at the time of diagnosis, but based on the literature, once her mutation has been identified and treatment has been started, her prognosis is favorable. The prognosis of treated cardiac amyloidosis has shown promising advancements in recent years, marking a significant stride in managing this complex and often challenging condition. The introduction of novel therapeutic approaches, including targeted medications and emerging technologies, as well as early detection and intervention, has improved outcomes for patients undergoing treatment and plays a crucial role in enhancing prognosis, as they allow for the timely implementation of disease-modifying therapies. Notably, partisiran was recently found to preserve functional capacity in patients with ATTR cardiac amyloidosis over 12 months of administration [7].

## Conclusions

Cardiac amyloidosis, a rare cause of restrictive cardiomyopathy, involves harmful amyloid protein deposition in the heart. Underdiagnosis is expected due to its subtle progression, requiring vigilant clinical suspicion and advanced imaging. This case study highlights the diagnostic challenges and delayed identification of transthyretin-related cardiac amyloidosis (ATTR) in an older adult. Non-invasive methods like echocardiography, cardiac magnetic resonance, and biomarker assays aid early detection. Multidisciplinary management involving cardiology and hematology focuses on heart failure, arrhythmia care, and disease-modifying agents. Genotyping guides treatment decisions. Raising awareness among healthcare professionals accelerates diagnosis and improves outcomes for patients with cardiac amyloidosis.
